# Emergence of *Neisseria meningitidis* ST-3587 harbouring *bla*_ROB-1_ and exhibiting dual resistance to penicillin and ciprofloxacin, Spain, 2024

**DOI:** 10.2807/1560-7917.ES.2026.31.4.2500398

**Published:** 2026-01-29

**Authors:** Josep Roca-Grande, Albert Moreno-Mingorance, Alba Bellés-Bellés, Joaquín Burgos, Jordi Càmara, Yannick Hoyos-Mallecot, Lucía López-Alcaide, Joan López-Madueño, Mayli Lung, Andrea Martín-Nalda, Alba Mir-Cros, Carmen Muñoz-Almagro, Amaresh Pérez-Argüello, Guillem Puigsech-Boixeda, M Dolores Quesada, Carolina Sarvisé, Aleix Soler-García, Pere Soler-Palacín, Jesús Trejo-Zahínos, Gloria Trujillo, Belén Viñado, M Nieves Larrosa, Juan José González-López

**Affiliations:** 1 Microbiology Research Group, Institut de Recerca Vall d’Hebron (VHIR), Hospital Universitari Vall d’Hebron Campus, Barcelona, Spain; 2 Department of Genetics and Microbiology, Universitat Autònoma de Barcelona (UAB), Cerdanyola del Vallès, Spain; 3 CIBER de Enfermedades Infecciosas (CIBERINFEC), Instituto de Salud Carlos III, Madrid, Spain; 4 Department of Clinical Microbiology, Hospital Universitari Arnau de Vilanova, Institut de Recerca Biomèdica de Lleida (IRB-Lleida), Lleida, Spain; 5 Department of Infectious Diseases, Hospital Universitari Vall d’Hebron Campus, Barcelona, Spain; 6 Infectious Diseases Research Group, Institut de Recerca Vall d’Hebron (VHIR), Barcelona, Spain; 7 Department of Medicine, Universitat Autònoma de Barcelona (UAB), Cerdanyola del Vallès, Spain; 8 Department of Clinical Microbiology, Hospital Universitari de Bellvitge, IDIBELL-UB, L’Hospitalet de Llobregat, Spain; 9 CIBER de Enfermedades Respiratorias (CIBERES), Instituto de Salud Carlos III, Madrid, Spain; 10 Department of Clinical Microbiology, Hospital Universitari Vall d’Hebron Campus, Barcelona, Spain; 11 Fundació Althaia, Xarxa Assistencial Universitària Manresa, Manresa, Spain; 12 Pediatric Infectious Diseases and Immunodeficiencies Unit, Children’s Hospital, Hospital Universitari Vall d’Hebron Campus, Barcelona, Spain; 13 Infectious Diseases and Microbiome Research Group, Institut de Recerca Sant Joan de Déu, Hospital Sant Joan de Déu, Esplugues, Spain; 14 CIBER de Epidemiología y Salud Pública (CIBERESP), Instituto de Salud Carlos III, Madrid, Spain; 15 School of Medicine and Health Sciences, Universitat Internacional de Catalunya (UIC), Barcelona, Spain; 16 Department of Clinical Microbiology, Hospital Universitari Germans Trias i Pujol, Universitat Autònoma de Barcelona (UAB), Badalona, Spain; 17 Department of Clinical Microbiology, Hospital Universitari de Tarragona Joan XXIII, Pere Virgili Health Research Institute (IISPV), Tarragona, Spain; 18 Department of Pediatrics, Hospital Sant Joan de Déu, Esplugues, Spain

**Keywords:** *Neisseria meningitidis*, ST-3587, antimicrobial resistance, ROB-1, β-lactamase, meningococcal urethritis

## Abstract

**BACKGROUND:**

Dual penicillin- and ciprofloxacin-resistant *Neisseria meningitidis* causing invasive meningococcal disease (IMD) have recently emerged in association with sequence type (ST) 3587, harbouring ROB-1 β-lactamase (*bla*_ROB-1_) and a mutated DNA gyrase (*gyrA*). These strains pose a threat to current antimicrobial treatment and prophylaxis.

**AIM:**

We aimed to characterise the first dual-resistant *N. meningitidis* ST-3587 isolates harbouring *bla*_ROB-1_ and a mutated *gyrA* identified in Spain.

**METHODS:**

Three *N. meningitidis* isolates encoding *bla*_ROB-1_ were identified in 2024. They were characterised by whole genome sequencing to determine capsular genogroups, ST and genetic antimicrobial resistance markers. Dated phylogenetic analysis was performed alongside global ST-3587 strains.

**RESULTS:**

The three *bla*_ROB-1_-encoding isolates belonged to ST-3587, genogroup Y, harboured a T91I mutation in *gyrA* and showed resistance to penicillin and ciprofloxacin. These isolates were obtained from urethral, oropharyngeal and blood samples, each from a different patient. According to the dated phylogenetic analysis of ST-3587 and the presence of *bla*_ROB-1_, two clades were defined: clade I and clade II. Within clade II, subclade II.I was identified, comprising isolates which, in addition to *bla*_ROB-1_, carried the T91I mutation in *gyrA*. This subclade included the three Spanish isolates, which exhibited close genetic relatedness.

**CONCLUSION:**

This study documents the emergence of *N. meningitidis* ST-3587 with dual resistance in Europe, including a documented urogenital infection by this lineage. Continued surveillance of antimicrobial resistance in *N. meningitidis*, including non-invasive cases, is crucial for timely public health responses and effective IMD prevention strategies.

**Key public health message**

**What did you want to address in this study and why?**

*Neisseria meningitidis*, also called meningococci, is a bacterium that healthy people can carry in their throat, but it can also cause severe invasive infection, especially in newborns and people with weakened immune systems. Some meningococci are becoming resistant to antibiotics. For this reason, we wanted to understand how these bacteria became resistant and have recently spread in Spain.

**What have we learnt from this study?**

Resistant meningococci were detected in Spain in 2024, in people affected by both bloodstream and genital infections. Genetic analysis showed that the strains identified in Spain were closely related and likely originated from a common source. They were probably imported at the same time from the Americas, where similar cases have been identified since the early 2010s.

**What are the implications of your findings for public health?**

*Neisseria meningitidis* is transmitted from person to person, often without causing any symptoms, which makes it hard to detect and control. The emergence of this bacterium as a sexually transmitted pathogen, together with the acquisition of antimicrobial resistance, highlights the need for close monitoring and for adapting prevention strategies when necessary.

## Introduction

*Neisseria meningitidis* is a Gram-negative diplococcus and a typical commensal of the human upper airway. This bacterium can cause invasive meningococcal disease (IMD), commonly presenting as meningitis and/or sepsis associated with high morbidity and mortality [1]. Treatment for IMD usually includes third generation cephalosporins (3GC), while prophylaxis includes rifampicin, ciprofloxacin or ceftriaxone. Other less common clinical manifestations of *N. meningitidis* infection are urogenital and anorectal infections, described in both males and females [2,3].

Nonsusceptibility to penicillin in *N. meningitidis* has increased globally over the past 30 years, with a marked increase in penicillin resistance since 2016 [4]. Penicillin resistance is primarily caused by mutations in *penA* that lead to the production of mosaic penicillin-binding protein 2 (PBP2), an essential cell wall peptidoglycan transpeptidase, or exceptionally by the production of the narrow spectrum β-lactamases *bla*_ROB-1_ and *bla*_TEM-1_ [4]. At least five critical residues (F504, A510, I515, H541 and I566) located in C-terminal of PBP2 are known to contribute to penicillin resistance [5]. Mosaic *penA* alleles also contribute to reduced effectiveness of other β-lactam antibiotics such as 3GC. Although rare, cases with cefotaxime-resistant *N. meningitidis* mediated by mosaic *penA* alleles probably acquired from other *Neisseria* species have recently been reported [6]. Quinolone-resistant isolates are rare in Europe [4]. However, in China, most isolates are resistant to ciprofloxacin (84%), mainly associated with clonal complex (cc) 4821 strains [7]. Ciprofloxacin resistance in *N. meningitidis* is primarily mediated through mutations in the quinolone resistance-determining region (QRDR) of the DNA gyrase gene (*gyrA*), generally affecting three residues (T91, D95 and T173), and the most frequently observed mutation is T91I [4].

A total of 33 cases of IMD caused by *N. meningitidis* producing ROB-1 β-lactamase were previously reported in the United States (US) between 2013 and 2020 [8]. Furthermore, six additional cases were reported in El Salvador in 2017–2019 [9]. Most isolates from the cases belonged to serogroup Y (NmY), sequence type (ST) 3587 and cc23. Eleven of the US isolates along with all six isolates from El Salvador were resistant to penicillin and ciprofloxacin and carried both *bla*_ROB-1_ and a T91I mutation in *gyrA*. The remaining 22 US isolates were resistant only to penicillin and carried *bla*_ROB-1_ alone [8,9]. However, these studies are limited to the American continent and were conducted before the COVID-19 pandemic. In Europe, only France reported the identification of an ST-3587 isolate harbouring *bla*_ROB-1_ in 2017 [10]. Overall, data on the circulation of ST-3587 isolates encoding *bla*_ROB-1_ in Europe, particularly in the post-pandemic period, are lacking. This is especially relevant considering the major disruptions the pandemic caused in the dynamics of nasopharyngeal pathobionts, including *N. meningitidis*, the circulation of which, and consequently the incidence of invasive disease, dropped significantly during 2020 and 2021 [11].

This study reports and characterises the first dual-resistant *N. meningitidis* ST-3587 isolates harbouring *bla*_ROB-1_ and a mutated *gyrA* identified in Spain, associated with IMD and meningococcal urethritis. Furthermore, we explore the phylotemporal evolution of this ST through whole genome sequencing (WGS).

## Methods

### Isolates and sociodemographic data collection

Three genogroup Y *N. meningitidis* isolates encoding the *bla*_ROB-1_ gene were recovered from clinical samples in Catalonia, Spain, in 2024. A comprehensive review of the patients’ medical records was performed to collect their clinical and sociodemographic characteristics.

### Whole genome sequencing and bioinformatic analysis

The isolates were sequenced and assembled as described previously [12]. Assemblies were submitted to the *Neisseria* spp. PubMLST database to characterise capsular genogroup, ST and cc [13]. Acquired genes involved in antimicrobial resistance were identified using the Resistance Gene Identifier (RGI) version 3.2.6 (https://card.mcmaster.ca/analyze/rgi). Alleles and amino acid mutations encoded in *penA* and *gyrA* were identified according to the PubMLST locus definition. Genome annotation was performed using Bakta version 1.9.4 (https://bakta.computational.bio/).

### Antimicrobial susceptibility testing

Antimicrobial susceptibility was determined using the gradient diffusion method (Etest, bioMérieux, Marcy-l'Étoile, France) for penicillin G, ceftriaxone, cefotaxime, meropenem, ciprofloxacin and rifampicin by incubating the isolates on Mueller-Hinton agar supplemented with 5% horse blood and 20 mg/L β-NAD (bioMérieux) for 18–24 h at 37°C in a 5% CO_2_ atmosphere. Minimum inhibitory concentrations (MICs) were interpreted according to the EUCAST 2024 clinical breakpoint values (https://eucast.org/).

### Dated phylogenetic analysis

All *N. meningitidis* ST-3587 records in the PubMLST database were screened in January 2025 to identify isolates with available raw sequence data (FASTQ files) in the National Center for Biotechnology Information (NCBI) database. Supplementary Table S1 lists all the ST-3587 sequences retrieved from the NCBI database, along with their accession numbers and metadata. When available, FASTQ files were retrieved, and along with the three isolates sequenced in this study, they were used to perform a dated phylogenetic analysis and to provide context on the global circulation of ST-3587. FASTQ files were filtered using Trimmomatic version 0.39 (https://github.com/timflutre/trimmomatic) and the resulting files were used to calculate genetic distances based on single nucleotide polymorphism (SNP) analysis with Snippy version 4.3.6 (https://github.com/tseemann/snippy), using the complete genome of *N. meningitidis* strain 11–7 (serogroup Y, ST-23, cc23; GenBank accession number CP021520.1) as reference. Strain 11–7 was selected as a representative of cc23 that does not encode the *bla*_ROB-1_ gene. Recombination regions were identified and removed using Gubbins version 2.4.1 [14]. The output generated by Gubbins, along with the sampling date for each isolate (or the year range when the sampling date was not available), was used to construct a Bayesian dated phylogeny using BactDating version 1.1.2 (https://xavierdidelot.github.io/BactDating/reference/BactDating.html).

Four molecular clock models (Poisson, Negative Binomial, Mixed Gamma and Mixed Continuous Additive Relaxed Clock) were performed with 10^7^ iterations each to ensure that the effective sample size of the inferred parameters exceeded 200 in the Markov chain Monte Carlo analysis. The model with the lowest deviance information criterion (Poisson model) was used for the final analysis. Significance of clock signal was tested by running the algorithm again with all sampling dates forced equal under the same conditions (Poisson model with 10^7^ iterations) and concluded that the temporal signal was significant. The estimated date of the most recent common ancestor (MRCA) and its 95% highest posterior density (HPD) were obtained directly from the BactDating output, inferred based on genetic divergence and sampling dates. The MRCA was defined as the last shared ancestor of all sequences within a defined group, and the dates when common ancestors are estimated to have existed are represented by the internal nodes [15].

## Results

### Bacterial isolates and characteristics of patients

The three isolates (H15-GEN-003, H15-RESP-032 and H15-EMI-061) encoding *bla*_ROB-1_ were obtained from three adult patients living in the same county in 2024. The first isolate was recovered from a patient with suspected urethritis; the second from a female patient who had sexual contact with a male partner presenting urethritis; and the third from a patient presenting to the emergency room with bacteraemia (Table 1). All three isolates harboured an intact genogroup Y capsule locus and shared the same molecular profile: P1.5–2,10–2:F4–1:ST-3587:cc23.

**Table 1:** Clinical and epidemiological characteristics of cases with *Neisseria* *meningitidis* isolates encoding *bla*_ROB-1,_ Spain, 2024 (n = 3)

Identification code	Case description
H15-GEN-003	The isolate was recovered from a urethral swab of an adult male of Spanish origin presenting dysuria and mild urethral discharge, referring to recent oral sex practices. Testing for other commonly sexually transmitted pathogens was negative^a^. Symptoms resolved after treatment with ceftriaxone and doxycycline.
H15-RESP-032	The isolate was recovered from a throat swab of an asymptomatic adult female of Latin American origin who consulted following recent oral sexual practices with a male partner presenting urethritis. Testing for other commonly sexually transmitted pathogens was negative^a^. The patient received no treatment.
H15-EMI-061	The isolate was recovered from a blood culture of an adult female of Latin American origin residing in Spain for months. The patient presented fever accompanied by chills, generalised arthromyalgia, cephalalgia, asthenia, anorexia and liquid diarrhoea without vomiting. The IMD episode resolved after treatment with ceftriaxone.

IMD: invasive meningococcal disease.

^a^ Tested for *Neisseria gonorrhoeae*, *Chlamydia trachomatis*, *Mycoplasma genitalium* and *Trichomonas vaginalis*.

### Antimicrobial susceptibility testing

Antimicrobial susceptibility testing showed that the three isolates were resistant to penicillin and ciprofloxacin and susceptible to cefotaxime, ceftriaxone, meropenem and rifampicin (Table 2). The chromosomal region containing the *bla*_ROB-1_ insert was identical across the three isolates and matched that previously described in the French isolate (data not shown) [10]. In addition to *bla*_ROB-1_, the three isolates carried the mosaic *penA*9 allele, which encoded the five characteristic substitutions F504L, A510V, I515V, H541N and I566V in PBP2, associated with penicillin resistance in *N. meningitidis* [5]. Regarding ciprofloxacin resistance, isolates presented the T91I mutation in the QRDR of *gyrA* (*gyrA*242 allele).

**Table 2:** Antimicrobial susceptibility testing results of *Neisseria meningitidis* isolates, Spain, 2024 (n = 3)

Antimicrobial agent	MIC (μg/mL)		
	H15-GEN-003	H15-RESP-032	H15-EMI-061
Penicillin G	**8**	**8**	**8**
Cefotaxime	0.012	0.008	0.006
Ceftriaxone	< 0.016	< 0.016	< 0.016
Meropenem	0.047	0.032	0.032
Ciprofloxacin	**0.19**	**0.19**	**0.19**
Rifampicin	0.023	0.016	0.032

MIC: minimum inhibitory concentration.

MICs higher than the clinical breakpoint are indicated in bold.

MICs were interpreted according to the EUCAST 2024 clinical breakpoint values (https://eucast.org/ ).

### Genomic epidemiology of *Neisseria meningitidis* ST-3587

To gain insight into the ST-3587 *N. meningitidis* population, a dated phylogenetic analysis was performed using the three isolates sequenced in this study, along with all publicly available raw sequence data (FASTQ files) of *N. meningitidis* ST-3587 isolates retrieved from the NCBI database (n = 39). According to the presence of *bla*_ROB-1_, we defined two clades within the dated phylogenetic tree (Figure).

**Figure:**
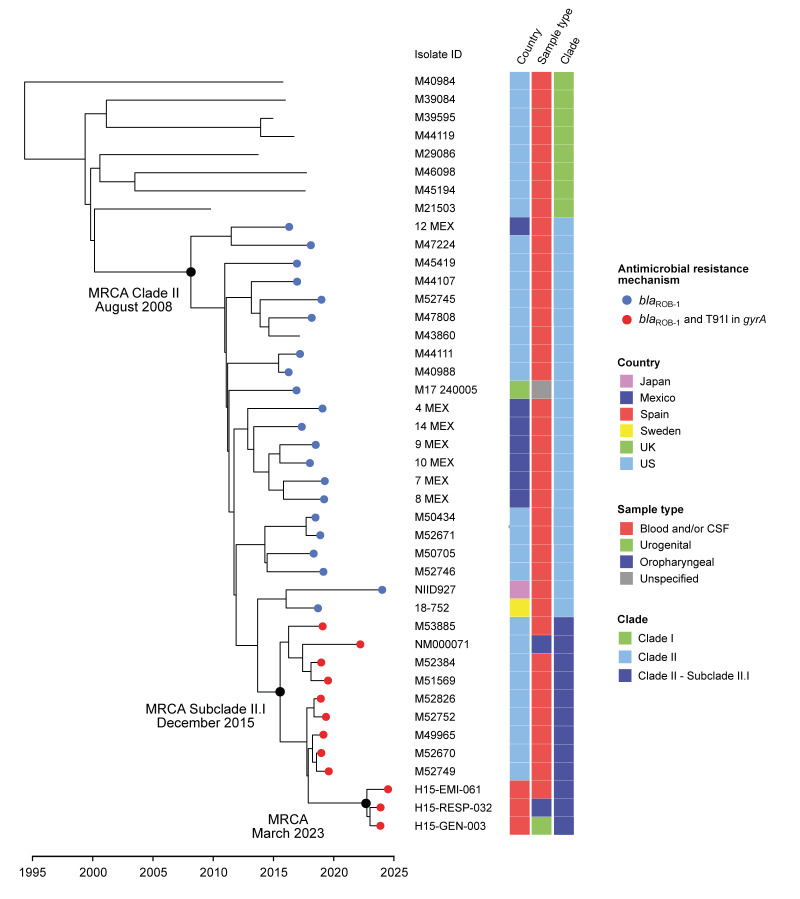
Time-scaled phylogeny of *Neisseria meningitidis* isolates belonging to ST-3587, Spain (n = 3) and globally (n = 39), 2010–2024^a^

CSF: cerebrospinal fluid; ID: identification code; MRCA: most recent common ancestor; ST: sequence type; UK: the United Kingdom; US: the United States.

Coloured dots at the end of the tree branches indicate isolates harbouring *bla*_ROB-1_. Identifier, country, sample type, and clade are also indicated for each isolate on the right side.

^a^ The global isolates were from NCBI (https://www.ncbi.nlm.nih.gov).

Clade I comprised eight isolates from the US obtained between 2010 and 2018, all of which were recovered from IMD cases. None of these isolates harboured *bla*_ROB-1_ or the T91I mutation in *gyrA*. Clade II included 33 isolates recovered between 2016 and 2024, all but one of which harboured *bla*_ROB-1_. Within this clade, subclade II.I was identified, consisting of *N. meningitidis* isolates that, in addition to *bla*_ROB-1_, possessed the T91I mutation in *gyrA*. This subclade comprised eight isolates obtained in 2019, one isolate from 2022, and the three Spanish isolates described in this study, obtained in 2024. Most isolates within clade II originated from the US, but isolates from Mexico, Sweden, the United Kingdom (UK) and Japan were also present. Most isolates were recovered from IMD cases, except for one UK isolate of unknown origin and one US isolate recovered from an oropharyngeal carrier.

The dated phylogeny analysis estimated the time to the MRCA of the clade II isolates to be August 2008 (95% HPD: February 2006–July 2010). The time to the MRCA of subclade II.I was estimated to be December 2015 (95% HPD: September 2014–November 2016). The three Spanish isolates formed a small cluster with a pairwise SNP distance range of 0–2, with the MRCA estimated to be March 2023 (95% HPD: July 2022–September 2023).

## Discussion

In this study, we report the detection and spread in Spain of *N. meningitidis* harbouring *bla*_ROB-1_ exhibiting dual resistance to both penicillin and ciprofloxacin. Genomic analysis revealed that the isolates belonged to genogroup Y and ST-3587, which have previously been identified in North and Central America, where several IMD cases have been reported [8,9]. Additionally, we report the potential of ST-3587 isolates to cause urogenital infections.

To our knowledge, one report has documented the presence of an ST-3587 isolate in Europe, specifically in France in 2017, which also carried *bla*_ROB-1_ but lacked the T91I mutation in *gyrA* [10]. Given that two of the three patients in this study were of Latin American origin and that these β-lactamase-producing strains have previously been associated with individuals of Hispanic ethnicity in the US [16], their emergence in Spain is more likely the result of introduction from the American continent, and subsequent local dissemination, rather than dissemination from neighbouring European countries. Notably, the patient from whom H15-EMI-061 was obtained had recently relocated to Spain from a Latin American country, further supporting a potential importation route from America. More recently, this ST was also reported in Japan in 2024 [17]. However, our findings reveal that the isolates identified in this study and those reported in Japan represent divergent genetic evolutionary paths, as evidenced by their separation into distinct branches and their placement within two distinct clades in the dated phylogenetic tree. These results suggest that both ST-3587 isolates, with and without the *gyrA* T91I mutation, are currently co-circulating.

The MRCA of all the *bla*_ROB-1_ encoding ST-3587 isolates was estimated to have emerged in August 2008, which aligns with the identification of the earliest known *bla*_ROB-1_ encoding ST-3587 isolate in 2013 [8], giving rise to clade II. As previously hypothesised, *bla*_ROB-1_ may have been acquired through horizontal gene transfer (HGT) during respiratory co-colonisation with *Haemophilus influenzae* carrying the pB1000’ plasmid [10,18]. Following this initial acquisition, the ST-3587 continued to circulate until a second evolutionary event occurred, marked by the acquisition of the *gyrA*242 allele containing the T91I mutation conferring resistance to ciprofloxacin, which likely occurred through HGT from *Neisseria lactamica* [8]. This acquisition was estimated to have occurred in December 2015, giving rise to subclade II.I. The first ST-3587 isolates exhibiting dual resistance to both penicillin and ciprofloxacin were identified in El Salvador in 2017 [9], consistent with the estimated MRCA. Until 2020, isolates from both clade II and subclade II.I had been detected circulating in the US [8]. As mentioned previously, the present study revealed that subclade II.I isolates had recently been introduced into Spain, while ciprofloxacin-susceptible clade II isolates had reached Japan [17]. Nevertheless, the possible circulation of these latter isolates in Spain cannot be disregarded. Therefore, continued genomic surveillance of *N. meningitidis* and antimicrobial resistance monitoring are essential to detect the emergence and spread of lineages that may compromise current treatment and prophylactic strategies.

Our findings also reveal the potential of ST-3587 isolates to cause urogenital disease. The non-invasive isolates H15-RESP-032 and H15-GEN-003 identified in this study showed a genetic distance of 0 SNPs. These isolates were collected within a 1-week interval in the same municipality. The patients from whom the isolates were obtained reported a single sexual partner each within the previous 3 months and recent sexual practices that resulted in symptoms of urethritis in the male partner. Additionally, no other sexually transmitted pathogens were identified. Recently, *N. meningitidis* has been isolated from urethral samples in men with urethritis [19,20]. Furthermore, its ability to cause urogenital infections has been described previously, including meningococcal urethritis caused by serogroup Y isolates [21-23], supporting the hypothesis of a recent acquisition through the same transmission chain between both patients, presumably via sexual contact, resulting in meningococcal urethritis. Regarding invasive isolate H15-EMI-061, although it was collected in the same county and showed only 2 SNPs of genetic distance from the others, no epidemiological link could be established between the cases. Phylogenetic analysis further revealed that this isolate did not descend from the first two. Consequently, the genetic and epidemiological evidence suggest that H15-EMI-061 did not result from onward transmission from the initial cases. These findings point to the existence of an undetected transmission chain that may have contributed to the dissemination of these strains. In this context, individuals not captured in the current epidemiological investigation, such as visiting friends and relatives, could have played a role in the spread of this ST.

These findings highlight the potential for local transmission of antimicrobial-resistant *N. meningitidis* lineages, which may compromise current treatment and prophylaxis. In this context, vaccination plays a critical role in disease prevention and transmission control. In Spain, the MenACWY conjugate vaccine was incorporated into the national immunisation programme schedule in 2020, replacing the monovalent MenC vaccine given at 12 years of age [24]. However, only some autonomous communities, including Catalonia in 2023 [25], have extended this replacement to the 12-month vaccination schedule, which could be advisable considering the need to reduce meningococcal transmission and protect high-risk populations. Importantly, while the MenACWY vaccine provides protection against serogroup Y, its effectiveness in certain high-risk populations remains limited, such as patients with weakened immune systems and those with complement deficiencies, among others. For instance, patients receiving eculizumab may remain vulnerable to invasive disease despite vaccination; therefore, continuous antimicrobial prophylaxis with penicillin is recommended for the duration of treatment in these individuals [26]. For this reason, vaccination of key target population, including infants, is advised to reduce oropharyngeal carriage [27] and thereby limit the dissemination of ST-3587 and other resistant lineages that could undermine prophylaxis strategies, especially in vulnerable groups.

The main limitation of this study is that not all globally identified *N. meningitidis* ST-3587 isolates could be included in the dated phylogenetic analysis, as raw sequence data (FASTQ files) were not available for all ST-3587 isolates listed in the PubMLST database. The inclusion of these missing isolates could improve the resolution and robustness of the phylogenetic analysis, helping to better elucidate the global dissemination patterns of this lineage.

## Conclusion

We documented the identification of *N. meningitidis* ST-3587 harbouring *bla*_ROB-1_ and exhibiting dual resistance to penicillin and ciprofloxacin in Spain and Europe, including a case of meningococcal urethritis caused by this ST. These findings highlight the emerging urogenital transmission of *N. meningitidis* and its increasing acquisition of antimicrobial resistance determinants. Ongoing surveillance, including non-invasive cases, is recommended to ensure that IMD prevention strategies, whether vaccination or antimicrobial prophylaxis, are adapted to the local epidemiological situation.

## Data Availability

The genome assembly sequences used in this study were deposited in the PubMLST *Neisseria* spp. database. The identification numbers for isolates are provided in Supplementary Table S1. Additionally, raw sequence files were deposited in the NCBI database under project accession no. PRJNA1268441.

## Supplementary Data

122000 Supplementary Material
