# Supplementary material for: Emergence of Neisseria meningitidis ST-3587 harbouring bla ROB-1 and exhibiting dual resistance to penicillin and ciprofloxacin, Spain, 2024
Source: Euro Surveill. 2026 Jan 29;31(4):2500398. doi: 10.2807/1560-7917.ES.2026.31.4.2500398 (PMC12859395; doi:10.2807/1560-7917.ES.2026.31.4.2500398)
Supplement: Supplementary Material [file 25-00398_GONZALEZ-LOPEZ_Supplement.pdf]

Supplementary Material

This supplementary material is hosted by *Eurosurveillance* as supporting information alongside the article "Emergence of *Neisseria meningitidis* ST-3587 harbouring bla_{ROB-1} and exhibiting dual resistance to penicillin and ciprofloxacin, Spain, 2024", on behalf of the authors, who remain responsible for the accuracy and appropriateness of the content. The same standards for ethics, copyright, attributions and permissions as for the article apply. Supplements are not edited by *Eurosurveillance* and the journal is not responsible for the maintenance of any links or email addresses provided therein.

Supplementary Table S1. List of *N. meningitidis* ST-3587 genomes with raw sequence files available at the NCBI database, including the three Spanish isolates from this study.

PubMLST ID	Alias	Run Accession no.	Country	Year of isolation	Disease	Genogroup	ST	*bla*$_{ROB-1}$	T91I (gyrA)	Clade
53207	M17 240005	ERR1994481	UK	2017	Unk	NG	3587	Yes	No	Clade II
85265	18-752	ERR11181318	Sweden	2018	IMD	Y	3587	Yes	No	Clade II
91773	4 MEX	ERR3486031	Mexico	2019	IMD	Y	3587	Yes	No	Clade II
91775	7 MEX	ERR3486033	Mexico	2019	IMD	Y	3587	Yes	No	Clade II
91776	8 MEX	ERR3486034	Mexico	2019	IMD	Y	3587	Yes	No	Clade II
91777	9 MEX	ERR3486035	Mexico	2018	IMD	Y	3587	Yes	No	Clade II
91778	10 MEX	ERR3486036	Mexico	2018	IMD	Y	3587	Yes	No	Clade II
91780	12 MEX	ERR3486038	Mexico	2016	IMD	Y	3587	Yes	No	Clade II
91782	14 MEX	ERR3486040	Mexico	2017	IMD	Y	3587	Yes	No	Clade II
96233	M21503	SRR8200215	USA	2010	IMD	Y	3587	No	No	Clade I
96240	M29086	SRR8200457	USA	2014	IMD	Y	3587	No	No	Clade I
96242	M39084	SRR23157268	USA	2016	IMD	Y	3587	No	No	Clade I
96243	M39595	SRR23157595	USA	2015	IMD	Y	3587	No	No	Clade I
96244	M40984	SRR23157473	USA	2016	IMD	Y	3587	No	No	Clade I
96245	M40988	SRR23157469	USA	2016	IMD	Y	3587	Yes	No	Clade II
96248	M43860	SRR23157420	USA	2017	IMD	Y	3587	No	No	Clade II
96249	M44107	SRR23157415	USA	2017	IMD	Y	3587	Yes	No	Clade II
96250	M44111	SRR23157832	USA	2017	IMD	Y	3587	Yes	No	Clade II
96251	M44119	SRR23157825	USA	2017	IMD	Y	3587	No	No	Clade I
96253	M45194	SRR23157401	USA	2018	IMD	Y	3587	No	No	Clade I
96254	M45419	SRR23157399	USA	2017	IMD	Y	3587	Yes	No	Clade II
96255	M46098	SRR23157189	USA	2018	IMD	Y	3587	No	No	Clade I
96257	M47224	SRR23157388	USA	2018	IMD	Y	3587	Yes	No	Clade II

96258	M47808	SRR23157680	USA	2018	IMD	Y	3587	Yes	No	Clade II
96260	M49965	SRR23157767	USA	2019	IMD	Y	3587	Yes	Yes	Subclade II.I
96263	M50434	SRR23157750	USA	2018	IMD	Y	3587	Yes	No	Clade II
96266	M50705	SRR23157977	USA	2018	IMD	Y	3587	Yes	No	Clade II
96267	M51569	SRR23157627	USA	2019	IMD	Y	3587	Yes	Yes	Subclade II.I
96269	M52384	SRR23157720	USA	2019	IMD	Y	3587	Yes	Yes	Subclade II.I
96270	M52670	SRR23157712	USA	2019	IMD	Y	3587	Yes	Yes	Subclade II.I
96271	M52671	SRR23157711	USA	2019	IMD	Y	3587	Yes	No	Clade II
96272	M52745	SRR23157615	USA	2019	IMD	Y	3587	Yes	No	Clade II
96273	M52746	SRR23157614	USA	2019	IMD	Y	3587	Yes	No	Clade II
96274	M52749	SRR23157931	USA	2019	IMD	Y	3587	Yes	Yes	Subclade II.I
96275	M52752	SRR23157928	USA	2019	IMD	Y	3587	Yes	Yes	Subclade II.I
96276	M52826	SRR23157918	USA	2019	IMD	Y	3587	Yes	Yes	Subclade II.I
96280	M53885	SRR23157902	USA	2019	IMD	Y	3587	Yes	Yes	Subclade II.I
115301	NM000071	SRR30496378	USA	2022	Carrier	Y	3587	Yes	Yes	Subclade II.I
148761	H15-GEN-003	SRR33711343	Spain	2024	Urethritis	Y	3587	Yes	Yes	Subclade II.I
154871	H15-RESP-032	SRR33711342	Spain	2024	Carrier	Y	3587	Yes	Yes	Subclade II.I
159976	NIID927	DRR610779	Japan	2024	IMD	Y	3587	Yes	No	Clade II
166510	H15-EMI-061	SRR33711341	Spain	2024	IMD	Y	3587	Yes	Yes	Subclade II.I

IMD: invasive meningococcal disease; NG: non-groupable; ST: sequence type; Unk: unknown; USA: United Sates of America; UK: United Kingdom
